# In vivo calcium imaging reveals disordered interictal network dynamics in epileptic *stxbp1b* zebrafish

**DOI:** 10.1016/j.isci.2021.102558

**Published:** 2021-05-19

**Authors:** Jing Liu, Kathryn A. Salvati, Scott C. Baraban

**Affiliations:** 1 Epilepsy Research Laboratory and Weill Institute for Neuroscience, Department of Neurological Surgery, University of California San Francisco, San Francisco, CA 94122, USA

**Keywords:** Optical imaging, Molecular neuroscience, Cellular neuroscience

## Abstract

*STXBP1* mutations are associated with encephalopathy, developmental delay, intellectual disability, and epilepsy. While neural networks are known to operate at a critical state in the healthy brain, network behavior during pathological epileptic states remains unclear. Examining activity during periods between well-characterized ictal-like events (i.e., interictal period) could provide a valuable step toward understanding epileptic networks. To study these networks in the context of *STXBP1* mutations, we combine a larval zebrafish model with *in vivo* fast confocal calcium imaging and extracellular local field potential recordings. *Stxbp1b* mutants display transient periods of elevated activity among local clusters of interacting neurons. These network “cascade” events were significantly larger in size and duration in mutants. At mesoscale resolution, cascades exhibit neurodevelopmental abnormalities. At single-cell scale, we describe spontaneous hyper-synchronized neuronal ensembles. That calcium imaging reveals uniquely disordered brain states during periods between pathological ictal-like seizure events is striking and represents a potential interictal biomarker.

## Highlights

• Calcium imaging in genetic epilepsy zebrafish model to study the interictal state
• Network cascade activity was more prominent in *stxbp1b* mutant zebrafish
• *stxbp1b* mutants characterized by spontaneous hyper-synchronized neuronal ensembles

## Graphical abstract

**Figure:**
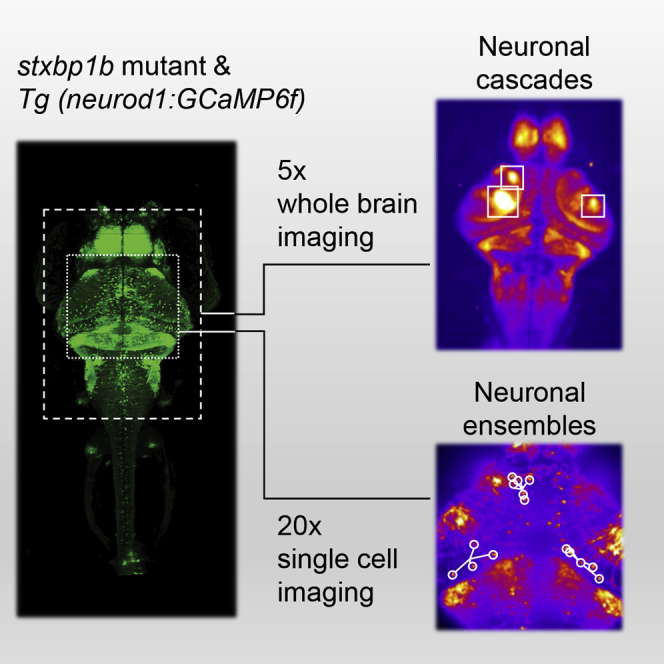

## Introduction

Network analysis of spatiotemporal patterns of brain activity is crucial to our understanding of normal, and pathological, brain states. Data from functional magnetic resonance imaging (fMRI), *in vitro* calcium imaging, local field potential (LFP), and/or multi-electrode array recordings have converged on an observation that neural networks operate at a dynamic balance between phases of order and disorder. Computational modeling based on these data and older theories derived from the study of avalanches (Paczuski et al., 1996), earthquakes (Gutenberg and Richter, 1954), nuclear chain reactions (Harris, 1989), or forest fires (Malamud et al., 1998) also postulates that our brain operates at the transition between these two phases (Beggs and Plenz, 2003; Massobrio et al., 2015; Shew and Plenz, 2012; Shew et al., 2009). Our brain displays network dynamics operating on the border between premature termination and uncontrolled explosive growth of neuronal activity. These brain states are accompanied by (i) transient, millisecond-duration periods of elevated activity among local clusters of interacting neurons, termed “avalanches” or “cascades” and (ii) longer, hundreds of seconds in duration, range temporal correlations in neuronal activity operating at slow time scales, perhaps representing a resting-state network (Priesemann et al., 2014; Shew and Plenz, 2012; Smit et al., 2011; Zhigalov et al., 2017). Cascades have been observed in acute slices of the rat cortex (Beggs and Plenz, 2003; Bellay et al., 2015; Shew et al., 2009), premotor and motor cortex in awake monkeys (Petermann et al., 2009), and visual cortex in anesthetized cats (Hahn et al., 2010). Surprisingly, brief neuronal cascades are not limited to more complex nervous systems and were recently observed by Ponce-Alvarez and colleagues using a brain-wide calcium imaging approach in wild-type larval transgenic zebrafish expressing genetically encoded calcium indicators (GCaMPs) (Ponce-Alvarez et al., 2018). Although studying these network phenomena *in vivo* under disease conditions is relatively rare, using magnetoencephalography techniques, Arviv et al. demonstrated that the brains of patients with adult refractory epilepsy were characterized by larger neuronal avalanches during interictal periods (Arviv et al., 2016). Whether similar patterns of network activity are present in a genetic form of epilepsy *in vivo* and at early stages of neurodevelopment is currently unknown.

Catastrophic epilepsies of childhood are defined by intractable unprovoked seizures, intellectual dysfunction, and behavioral disabilities. Many are associated with single gene mutations (Howard and Baraban, 2017). For example, childhood *STXBP1* (syntaxin-binding protein 1, also known as MUNC18-1) disorder is a haploinsufficiency associated with heterogeneous epilepsy phenotypes (Stamberger et al., 2016): early infantile epileptic encephalopathy (EIEE; also known as Ohtahara syndrome) (Saitsu et al., 2010; Tso et al., 2014), infantile spasms (also known as West syndrome) (Barcia et al., 2014; Otsuka et al., 2010), Lennox-Gastaut syndrome (Epi4K Consortium et al., 2013), and Dravet syndrome (Carvill et al., 2014). Additionally, *STXBP1* mutation can be associated with neurodevelopmental disorders without epilepsy (Hamdan et al., 2011; Stamberger et al., 2016). *STXBP1* loss-of-function mutations have been recapitulated in mice (Chen et al., 2020; Kovacevic et al., 2018; Miyamoto et al., 2017; Orock et al., 2018) and zebrafish (Grone et al., 2016). The latter exhibits spontaneous unprovoked electrographic seizures (i.e., definition of an epileptic condition), neurodevelopmental defects, and abnormal locomotor activity, recapitulating key phenotypes of human *STXBP1* encephalopathy. Larval zebrafish, with optical transparency, relatively small brain dimensions and well-established transgenic GCaMP-expressing lines (Chen et al., 2013), offer an ideal preparation for *in vivo* imaging of network dynamics (Ahrens et al., 2012; Dunn et al., 2016a; Muto et al., 2013; Thiele et al., 2014). This type of calcium imaging data provides a level of spatial and temporal resolution of network activity not possible with LFP recordings. Recent applications of this simple vertebrate model to epilepsy research made it possible to non-invasively monitor activity throughout the nervous system during a generalized seizure event (Diaz Verdugo et al., 2019; Liu and Baraban, 2019; Rosch et al., 2018; Turrini et al., 2017; Winter et al., 2017). However, previous imaging studies were limited to pharmacologically induced acute seizures, whereas combining GCaMP-expressing and *stxbp1b* mutant zebrafish lines allows us to study (for the first time) these dynamics in a genetic model of epilepsy.

Here, fast confocal calcium imaging was performed using *neurod1*:GCaMP-expressing *stxbp1b* mutant zebrafish larvae. Taking advantage of relatively low spontaneous ictal-like seizure event frequencies in homozygote *stxbp1b* mutant zebrafish, we focused our analysis on tectal network activity during non-ictal periods. Optic tectum is the most complex layered structure in larval zebrafish brain, and its cellular composition is morphologically diverse, incorporating both GABAergic inhibitory interneurons and glutamatergic excitatory projection neurons (DeMarco et al., 2020; Robles et al., 2011; Scott and Baier, 2009). Owing to a superficial location near the dorsal brain surface coupled with transparency of larval zebrafish and its crucial role in visual processing, optic tectum has been the focus of recent *in vivo* optical imaging studies (Antinucci et al., 2019; Barker and Baier, 2015; Bergmann et al., 2018; Dunn et al., 2016b; Heap et al., 2018; Henriques et al., 2019; Kramer et al., 2019; Thompson et al., 2016; Wang et al., 2019). At a mesoscale level, calcium activity resembling previously described neuronal cascades (Ponce-Alvarez et al., 2018; Scott et al., 2014; Tagliazucchi et al., 2012) was confirmed in the optic tectum of all larvae imaged here. Interestingly, *stxbp1b* mutant zebrafish were characterized by prominent and larger size cascade activity compared to age-matched controls. We also observed that cascades in *stxbp1b* mutants display significantly different neurodevelopmental trajectories compared to controls. Pharmacological blockade of gap junctions (GJs) significantly suppressed neuronal cascades in these mutants. Finally, at single-cell scale, epilepsy-related neuronal ensembles (Liu and Baraban, 2019; Truccolo et al., 2011, 2014) were also prominent in *stxbp1b* mutants, suggesting hyper-synchronization in local neural networks.

## Results

**Figure 1:**
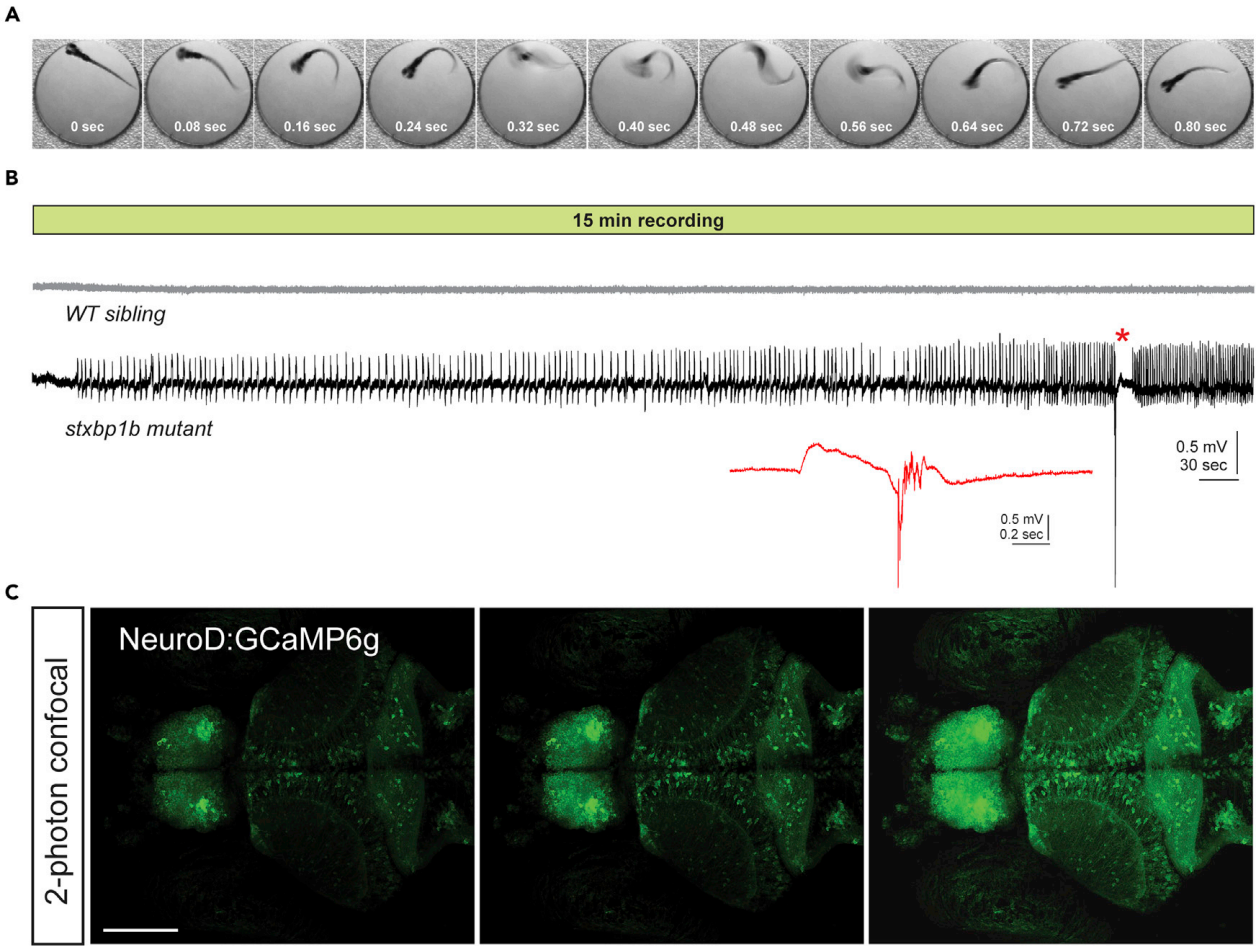
Characterization of epileptic phenotype in *stxbp1b* mutant zebrafish (A) Representative spontaneous high-velocity convulsive behavior captured during high-speed imaging (250 fps) of a single *stxbp1b* mutant larva freely swimming in embryo media at room temperature. Note these were rare events that occur with a velocity near, or greater than, the acquisition speed of the camera (QImaging Optimos cMOS). (B) Representative 15 min local field potential recordings from randomly selected larvae from a cross of *stxbp1b*^*+/-*^ adult breeders. LFP recordings were obtained from a glass microelectrode positioned under a microscope in the midbrain of agarose-embedded larvae at 5 dpf. Larvae were freed from the agarose and genotyped *post hoc*. Note the presence of small amplitude events building to a large amplitude multi-spike ictal event with postictal depression in the *stxbp1b* mutant larvae (see inset) but not the WT sibling. These recordings are representative of the spontaneous and unprovoked seizure activity associated with this zebrafish line. (C) Confocal images taken with a 2-photon microscope of a representative *neurod1*:GCaMP6f expressing larval zebrafish at three different levels through the central nervous system. Scale bar, 100 μm

We used CRISPR-Cas9-generated zebrafish carrying a 12 base-pair loss-of-function deletion in *stxbpb1b*, a brain expressed paralog sharing 79% amino acid sequence identity with human (Grone et al., 2016). Mutations in syntaxin-binding protein 1 (*STXBP1*) are a frequent cause of EIEE in humans (Stamberger et al., 2016). Homozygous *stxbp1b* mutant zebrafish larvae show phenotypic similarities to patients, including unprovoked convulsive-like behavior (Figure 1A) and spontaneous electrographic seizures (Figure 1B). Random monitoring of electrographic activity in *stxbp1b* larvae (5–7 days post fertilization [dpf]) using single electrode site LFP recording indicates a relatively low frequency of large amplitude ictal-like epileptiform discharges (1–2 events per 15 min recording in ∼20% of mutants; n = 28). Periods between ictal seizure events are classified as “interictal” and potentially relate to cognitive processing and epileptogenesis (Fisher et al., 2014; Shamshiri et al., 2019). In patients with epilepsy, interictal recordings are commonly used for presurgical evaluation and localization of epileptogenic brain regions (Lascano et al., 2012; Stefan et al., 2003; Wang et al., 2011). Here, we exploited the relatively low ictal-like seizure frequency in *stxbp1b* mutant larvae to study spontaneous neuronal activity and network dynamics during 5 min non-ictal imaging epochs.

### Prolonged cascades revealed in *stxbp1b* mutants by high-speed calcium imaging

**Figure 2:**
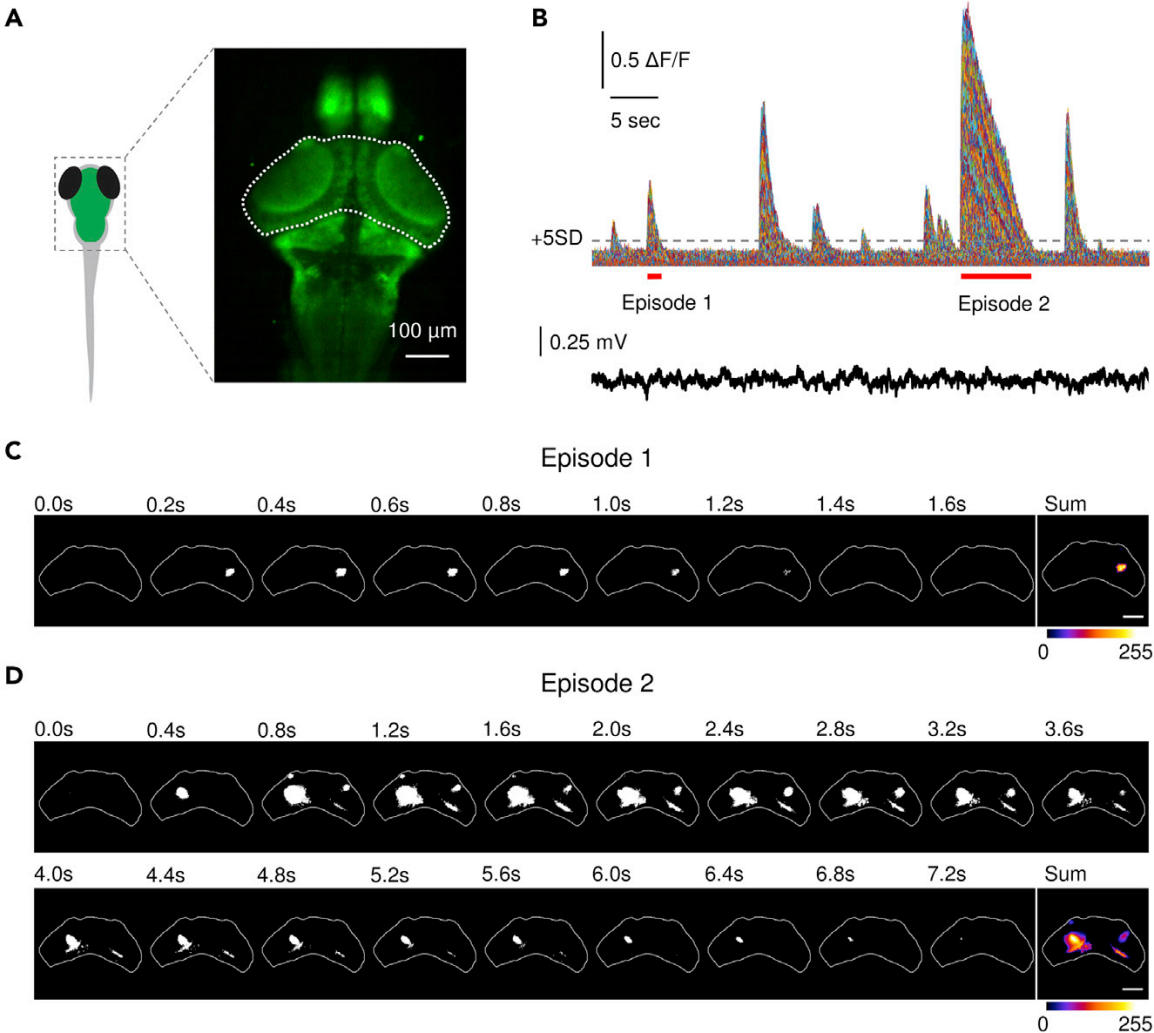
Measuring cascades in zebrafish optic tectum (A) Calcium imaging in *neurod1*:GCaMP6f-expressed larval zebrafish. Neuronal dynamics within optic tectum (highlighted by white dash line) were extracted for cascade measurement. (B) Calcium traces (ΔF/F) from pixels within the optic tectum in a *stxbp1b* mutant fish on 6 days post fertilization (dpf). A pixel is considered to be active when ΔF/F crosses the threshold of 5 times signal standard deviation (+5 SD). One pixel is 2.67 × 2.67 μm area. Simultaneously recorded local field potential (LFP) recording from midbrain is shown below. Scale bars as indicated in figure. (C and D) Binarized activity from episodes indicated in (B) (underlined in red). One normal cascade was revealed in (C), and multiple cascades were revealed in (D), including an abnormally large and long cascade located in the left optic tectum. The last frame represents the summation of the binary time series stack of the corresponding episode showing the spatial mapping of the cascades. The cumulative intensity of activation is color coded as shown in the color bar. Scale bars, 100 μm.

To study spatiotemporal patterns of brain activity during interictal periods, we performed blinded *in vivo* brain-wide imaging experiments in larval zebrafish (*stxbp1b*^*−/−*^ mutant and *stxbp1b*^*+/+*^ wild-type (WT) siblings; *stxbp1b*^*+/-*^ heterozygous data was excluded in this study) expressing *GCaMP6f* under the *neurod1* promoter (Rupprecht et al., 2016) (Figure 1C) at 5, 6, and 7 dpf. We used high-speed (20 fps) spinning disk confocal microscopy at mesoscale resolution (5x objective), focused on the optic tectum (comprising neuropil and stratum periventriculare; Figure 2A), to capture calcium activity clusters. As illustrated in Figure 2B, pixel fluorescence signals from optic tectum regions of interest were extracted and processed for analysis. We observed calcium activity ranging from brief small pixel coactivations (episode 1, Figure 2C; Video S1 [whole-brain imaging-WT]), which are ubiquitous in WT siblings (n = 36), to prolonged large coactivations (episode 2, Figure 2D; Video S2 [whole-brain imaging – mutant]), which are prominent in *stxbp1b* mutants (n = 36) but rarely seen in WT (see subsequent quantification analysis). Simultaneous LFP recordings (Figure 2B) failed to reveal any changes in the extracellular activity patterns at these single electrode sites consistent with a conclusion that cascades do not represent the widespread generalized activation of neurons seen during ictal-like seizures [see (Liu and Baraban, 2019)]. We defined these network phenomena wherein local clusters of spatially contiguous calcium activity patterns transiently emerge and then disappear as “cascades” (see STAR methods).

Video S1. Whole-brain imaging – WT Representative whole-brain imaging with 5× objective from WT sibling. Movie played at 10× speed. Related to Figure 2.

Video S2. Whole-brain imaging – mutant Representative whole-brain imaging with 5× objective from *stxbp1b* mutant, respectively. Movie played at 10× speed. Related to Figure 2.

**Figure 3:**
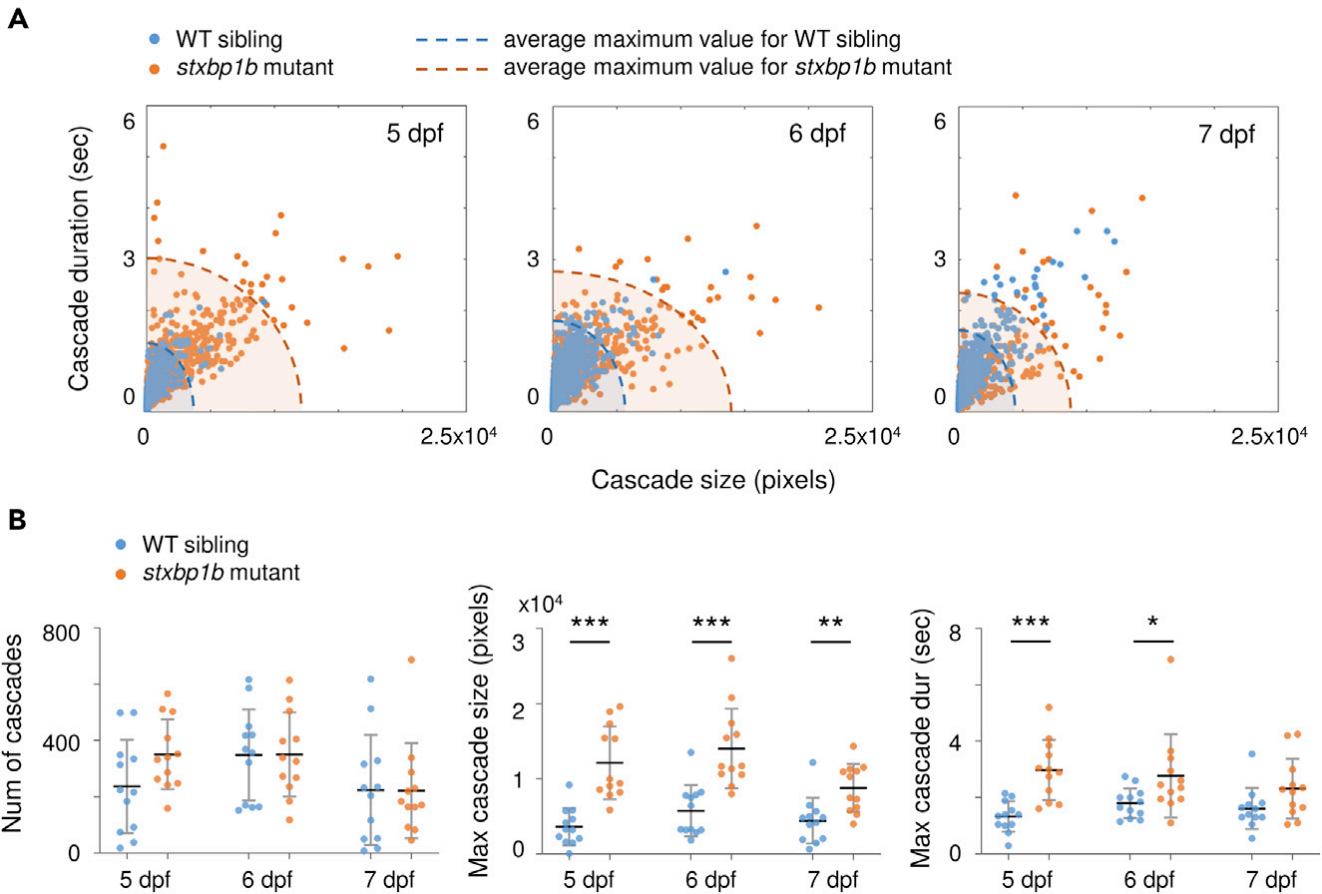
Cascade quantification (A) Cascade size versus duration on different days post fertilization (dpf). Blue dots represent WT siblings, and orange dots represent *stxbp1b* mutants. Dashed lines are the ellipse contour of the average maximum value of cascade size and duration (blue, WT siblings; orange, *stxbp1b* mutants). For WT siblings, n = 2843, 4188, and 2690 cascades on 5, 6, and 7 dpf, respectively. For *stxbp1b* mutants, n = 4212, 4214, and 2668 cascades on 5, 6, and 7 dpf, respectively. Data from 12 fish per day for each condition is plotted. (B) Probability distribution of cascade sizes on different dpf. The plots show the cumulative distributions of the corresponding data (blue, WT siblings; orange, *stxbp1b* mutants) on different dpf. Measurement of the number of cascade, maximum cascade size, and maximum cascade duration (per 5 min recording) on different dpf is shown. n = 12 fish per day for each condition. Data are represented as mean ± SD. Statistical significance is indicated as *∗*p < 0.05, *∗∗*p < 0.01, *∗∗∗*p < 0.001; Student's t test.

First, we characterized spatiotemporal patterns of cascades by calculating size, duration, and distribution. Figure 3A shows plots of cascade size versus duration; the most obvious difference between *stxbp1b* mutants and WT was seen at 5 dpf, where cascade size and duration of WT siblings are accumulated at small values, while mutant data are more scattered at large values. Interestingly, from 5 to 7 dpf, WT siblings showed an expanding contour of average maximum cascade size and duration, while *stxbp1b* mutants showed a shrinking contour. Second, we measured the number of cascades, maximum cascade size, and maximum cascade duration (Figure 3B). There was no significant difference in the number of cascades between *stxbp1b* mutants and WT siblings at any developmental ages (p = 0.0699, 0.9731, and 0.9806 for 5, 6, and 7 dpf, respectively, *t* test), but significantly larger cascades were seen in mutants at all three dpfs (p < 0.0001, p = 0.0002, and p = 0.0024 for 5, 6, and 7 dpf, respectively, *t* test), and significantly longer cascades were seen in *stxbp1b* mutants on 5 and 6 dpf (p < 0.0001, p = 0.0423, and p = 0.0706 for 5, 6, and 7 dpf, respectively, *t* test).

**Figure 4:**
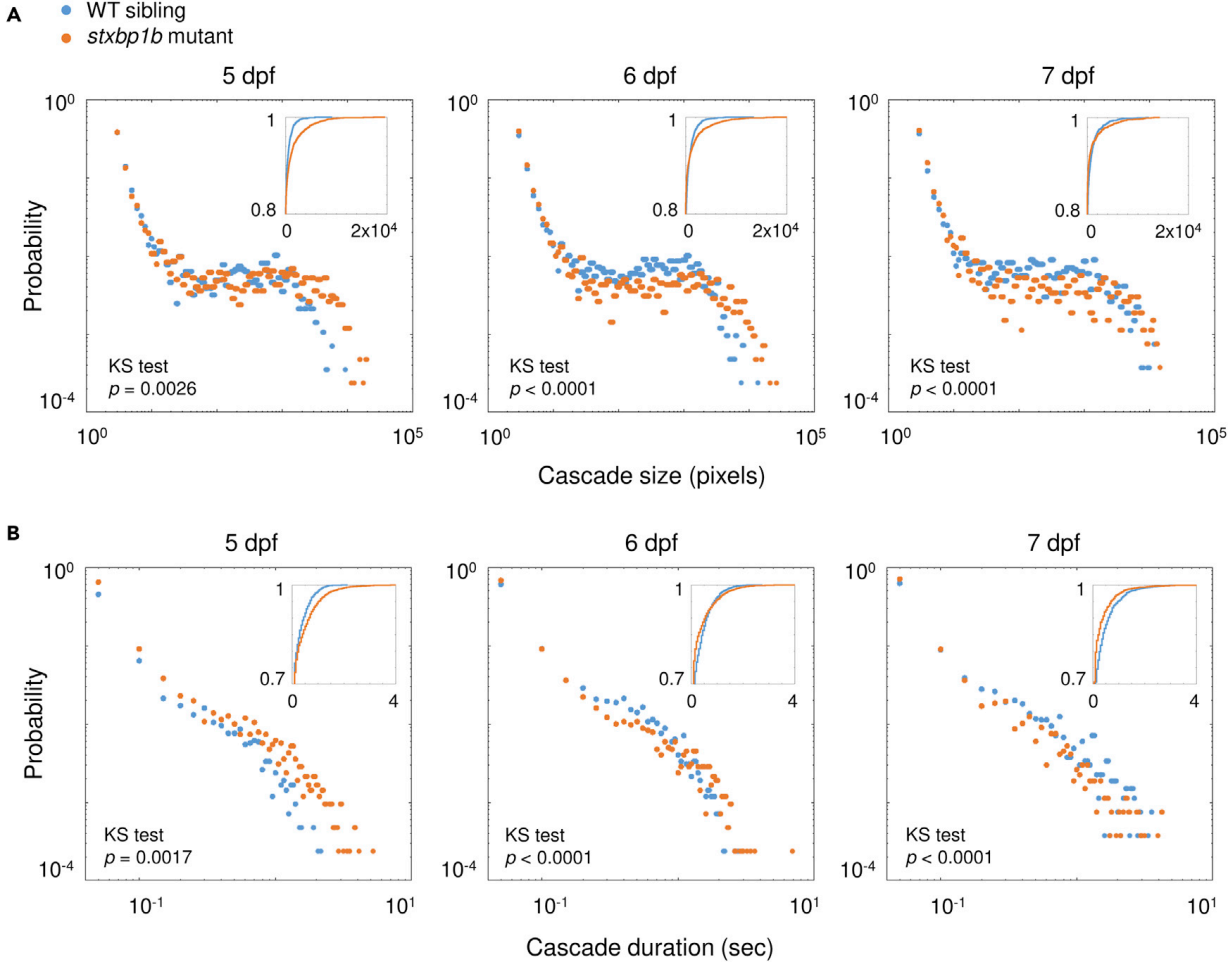
Probability distribution of cascade size and duration (A) Probability distribution of cascade sizes on different days post fertilization (dpf). The insert plots show the cumulative distributions of the corresponding data (blue, WT siblings; orange, stxbp1b mutants). (B) Probability distribution of cascade durations on different dpf. p values of KS tests are indicated in figure. Data from 12 fish per day for each condition is plotted.

Next, we examined the probability distribution of cascade size (Figure 4A). Significant divergence was noted between *stxbpb1* mutants and WT siblings on 5 and 6 dpf, where the mutant has a higher chance to show large cascades. The insert plots represent cumulative distribution of the corresponding data, with significant separation between mutants and WT siblings noted on large cascade probability at 5 dpf, on medium to large cascade probability at 6 dpf, and on medium cascade probability at 7 dpf (p = 0.0026, p < 0.0001, and p < 0.0001 for 5, 6, and 7 dpf, respectively, KS test). Similar results were seen in the probability distribution of cascade duration (Figure 4B). Significant divergences between mutants and WT siblings were observed on long cascade probability at 5 dpf and 6 dpf, where *stxbpb1* mutants have a higher chance to show prolonged cascades, and conversely, at 7 dpf, mutants have a lower chance to show long cascades (p = 0.0017, p < 0.0001, and p < 0.0001 for 5, 6, and 7 dpf, respectively, KS test).

**Figure 5:**
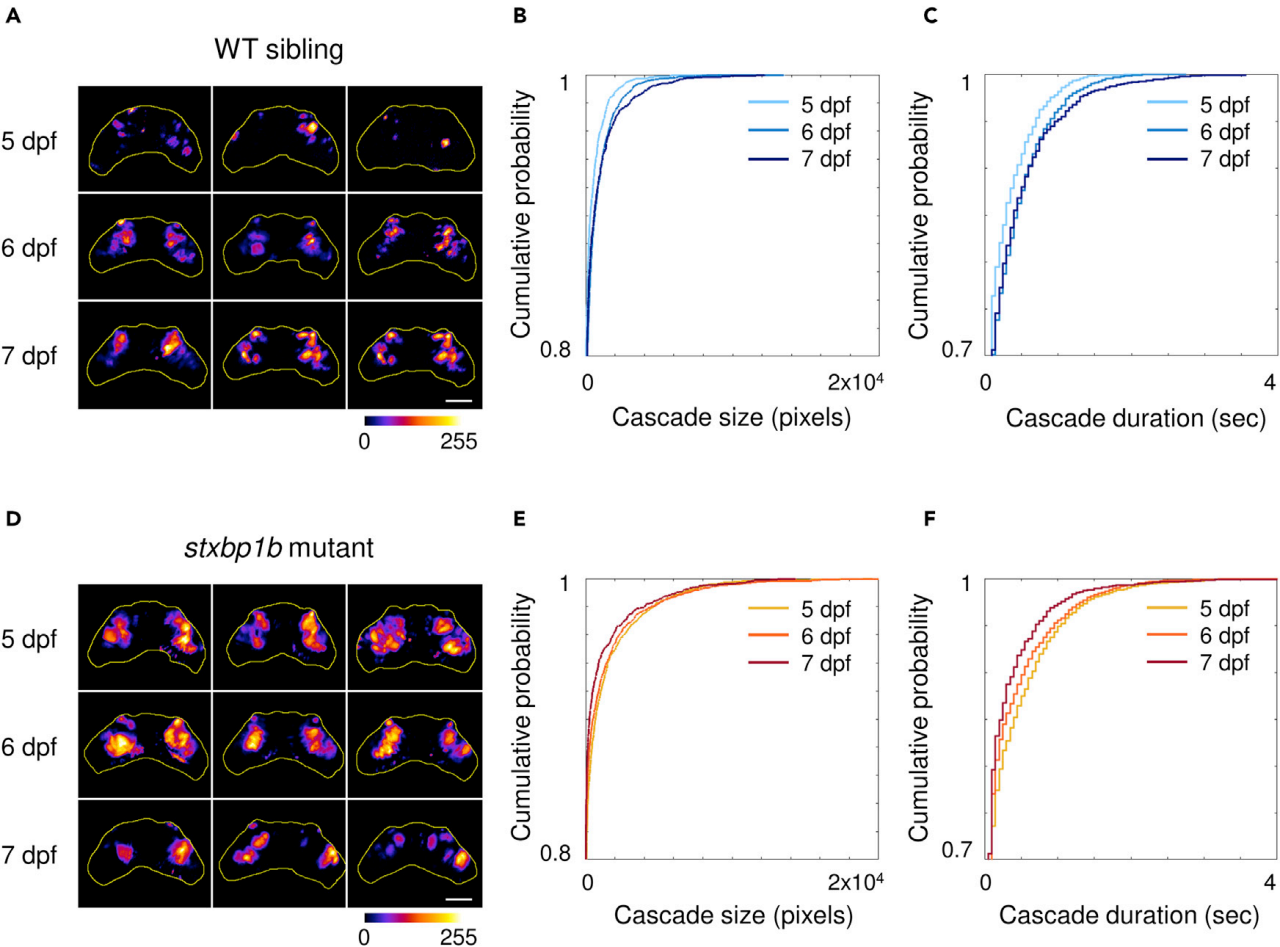
Cumulative distribution of cascade size and duration (A) Representative stack summation of binary time series from WT siblings on different days post fertilization (dpf). Each tile represents one fish. The cumulative intensity of activation is color coded as shown in the color bar. (B) Cumulative distribution of cascade sizes and durations from WT siblings on different dpf (light blue, 5 dpf; blue, 6 dpf; dark blue, 7 dpf). (C) Representative time series stack summation from *stxbp1b* mutants on different dpf. (D) Cumulative distribution of cascade sizes and durations from *stxbp1b* mutants on different dpf (yellow, 5 dpf; orange, 6 dpf; dark red, 7 dpf). Scale bars, 100 μm. Data from 12 fish per day for each condition is plotted.

Finally, with the same data set, we measured cumulative distribution of cascade size and duration. From 5 to 7 dpf, we can see a clear trend in WT siblings of increasing cascade size (Figure 5A; p < 0.0001, p = 0.4554, and p = 0.0001 for 5 dpf versus 6 dpf, 6 dpf versus 7 dpf, and 5 dpf versus 7 dpf, respectively, KS test) and duration (Figure 5B; p < 0.0001, p = 0.4554, and p = 0.0001 for 5 dpf versus 6 dpf, 6 dpf versus 7 dpf, and 5 dpf versus 7 dpf, respectively, KS test). Different from WT siblings, *stxbp1b* mutants showed the most prolonged large cascades on 5 dpf, and then, the cascade size and duration both declined at later ages (Figure 5C; for cascade size, p = 0.0006, p = 0.243, and p < 0.0001 for 5 dpf versus 6 dpf, 6 dpf versus 7 dpf, and 5 dpf versus 7 dpf, respectively, KS test; Figure 5D; for cascade duration, p = 0.0126, p = 0.083, and p < 0.0001 for 5 dpf versus 6 dpf, 6 dpf versus 7 dpf, and 5 dpf versus 7 dpf, respectively, KS test). Taken together, cascade changes observed at multiple levels of analysis suggest functional developmental differences in larval *stxbp1b* mutants compared to controls. This observation would be consistent with clinical classification of *STXBP1* as a “neurodevelopmental disorder” (Stamberger et al., 2016).

### Gap junctions play a role in cascade generation

**Figure 6:**
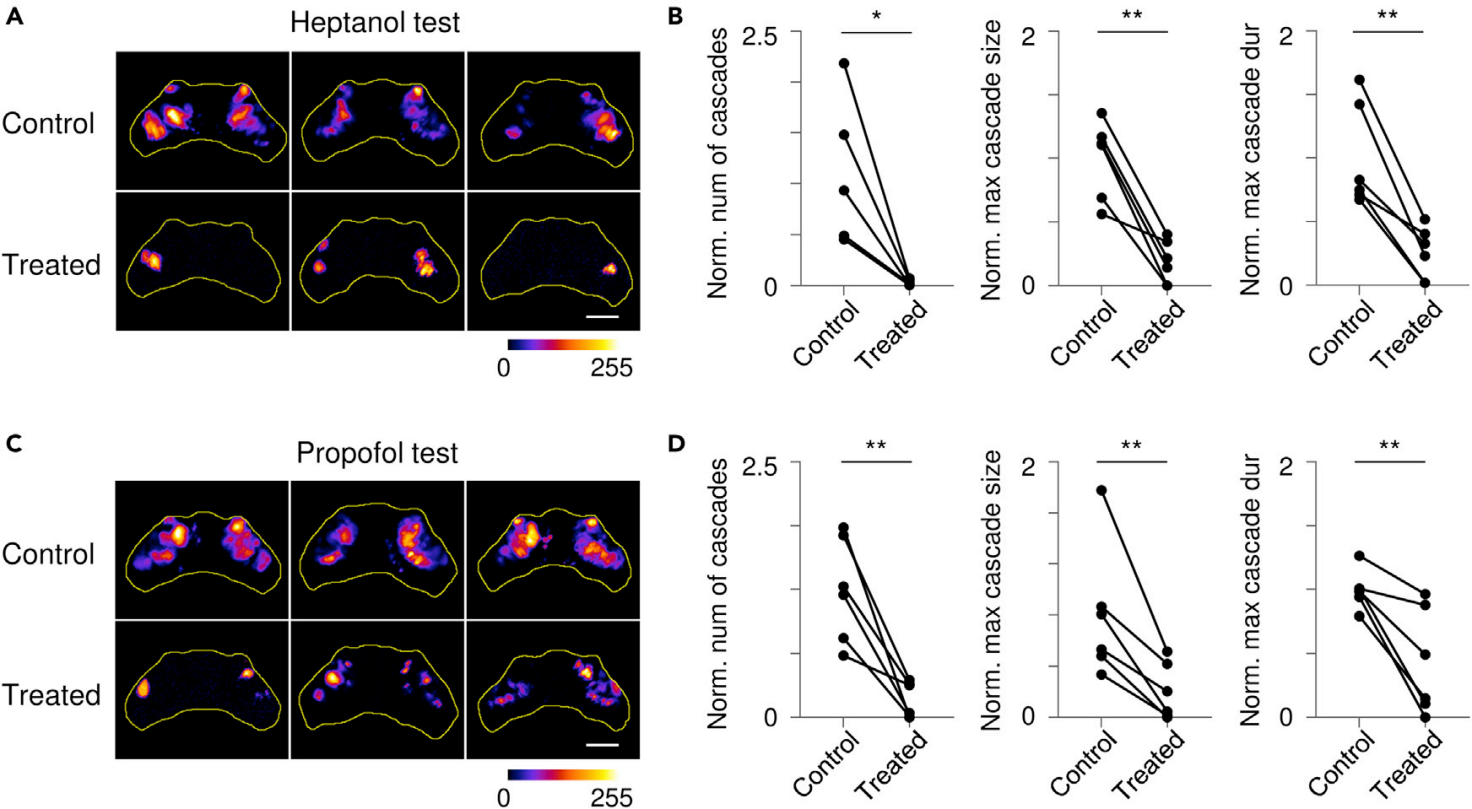
Gap junction blockers reduce neuronal cascade intensity (A) Representative stack summation of binary time series from *stxbp1b* mutants before (top row) and after (bottom row) heptanol treatment. The cumulative intensity of activation is color coded as shown in the color bar. (B) Comparison of normalized (norm.) number (num) of cascade, maximum (max) cascade size, and maximum cascade duration (dur) before and after heptanol treatment. Data were normalized to the average value from recordings before drug treatment. (C and D) Results from propofol experiments on *stxbp1b* mutants. Scale bars, 100 μm. n = 5 fish for each drug test. Statistical significance is indicated as *∗*p < 0.05, *∗∗*p < 0.01; Student's t test.

As there is growing evidence that a GJ network could play an important role in development of epilepsy (Patel et al., 2019; Robel and Sontheimer, 2016), we hypothesized that these interactions underlie local hyper-synchronization cascades observed in *stxbp1b* mutants. Because GJ interactions are largely mediated through electrical synapse communication (Giaume et al., 2010; Mylvaganam et al., 2014; Steinhäuser et al., 2012), we performed pharmacology studies using broad, well-established blockers: heptanol (Guan et al., 1997; Johnston et al., 1980; Weingart and Bukauskas, 1998) or propofol (Mantz et al., 1993; Wentlandt et al., 2006) (Figure 6). Ponce-Alvarez et al. recently reported that wild-type larvae exposed to heptanol display substantially fewer avalanche events (Ponce-Alvarez et al., 2018). We first assessed toxicity (1.5 hr incubation test) by monitoring heart activity in 3 separate agarose-embedded larvae at heptanol concentrations of 250 μM, 500 μM, and 1 mM and propofol concentrations of 10 μM, 25 μM, and 50 μM. At final concentrations of heptanol (500 μM) and propofol (10 μM), the resting heart rate could not be distinguished from pre-drug exposure control levels. However, when analyzing cascades, we noted that *stxbp1b* mutant larvae exposed to heptanol displayed a substantial reduction in cascade number (Figures 6A and 6B; p = 0.0177, paired *t* test; n = 6 mutants), maximum cascade size (p = 0.0016, paired *t* test), and maximum cascade duration (p = 0.003, paired *t* test). Data were normalized to the average value of non-treated period. Similar results were seen in propofol-exposed *stxbp1b* mutant larvae (Figures 6C and 6D; p = 0.0046, 0.0098, and 0.0078 for cascade number, maximum cascade number, and maximum cascade duration, respectively, paired *t* test; n = 6 mutants). These results indicate that pharmacological block of electrical synapses suppresses spontaneous cascades, suggesting a potential role for GJs.

### Epilepsy-related neuronal ensembles also revealed in *stxbp1b* mutants

**Figure 7:**
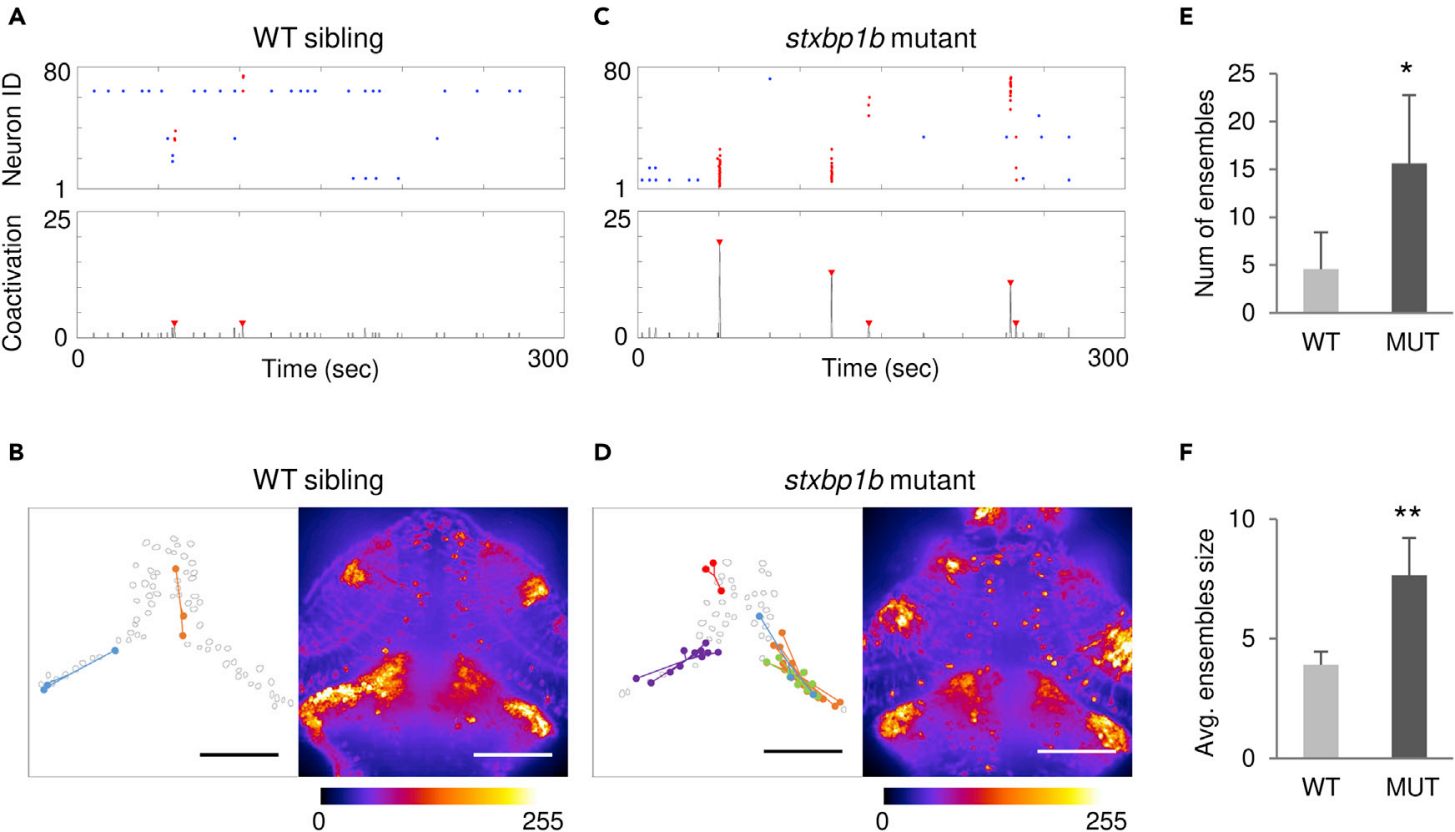
Neuronal ensembles revealed in *stxbp1b* mutants. (A) Representative neuron activation raster plot (top; red dots, ensemble neurons) and number of coactive neurons (bottom; ensembles were indicated by red arrow) from a WT sibling. (B) Left, spatial mapping of neuronal ensembles depicted in (A). Dots in the same color represent coactive neurons, and lines are distances from ensemble neurons to the corresponding ensemble centroid. Each ensemble is indicated by a color. Right, stack summation of calcium activity time series. The fluorescence intensity is color coded as shown in the color bar. (C and D) Representative data from a *stxbp1b* mutant. (E) Comparison of number (num) of ensembles. (F) Comparison of average (avg.) ensemble size. Scale bars, 100 μm. n = 5 fish each condition: for WT, 3, 1, and 1 fish from 5, 6, and 7 dpf, respectively; for mutants, 2, 2, and 1 fish from 5, 6, and 7 dpf, respectively. Data are represented as mean ± SD. Statistical significance is indicated as *∗*p < 0.05, *∗∗*p < 0.01; Student's t test.

Intracranial recordings in patients with epilepsy revealed “microseizure” discharges from spatially restricted neuronal populations (Schevon et al., 2008, 2010; Stead et al., 2010). These spatial differences in neuronal coactivation may be a functional feature distinguishing an epileptic brain state. Using fast confocal calcium imaging of individual neurons in larval zebrafish optic tectum, we previously identified ensembles of coactive neurons during interictal periods in an acute chemoconvulsant seizure model (Liu and Baraban, 2019). Here, we adapted this same strategy to analyze network dynamics in optic tectum at single-cell microscale resolution (20x objective) using a genetic model of epilepsy featuring spontaneous seizures (Grone et al., 2016). Network dynamics of optic tectum microcircuits are exemplified in Video S3 (optic tectum imaging – WT) and Video S4 (optic tectum imaging – mutant) for WT siblings and *stxbp1b* mutants, respectively. We constructed raster plots of neuronal activity from fluorescence changes (top panel in Figures 7A and 7C) with an automatic event detection algorithm (see STAR methods) and then used a sliding window technique to generate a coactive neuron number time series. Ensemble events, defined as a statistically significant number of coactive neurons compared with surrogate data sets, are marked by red arrowheads (bottom panel in Figures 7A and 7C), and corresponding coactive neurons are colored red in the raster plot. Representative spatial mapping of these ensembles onto the optic tectum is shown in Figures 7B and 7D. In the representative stack summation images, expanded activation clusters were observed in neuropil regions of *stxbp1b* mutants. We then quantified ensemble occurrence and average size, i.e., number of coactive neurons within an ensemble (Figures 7E and 7F). Consistent with earlier observations of neuronal ensembles in zebrafish during exposure to the chemoconvulsant pentylenetetrazole (PTZ) (Liu and Baraban, 2019), epileptic *stxbp1b* mutants, compared with WT siblings, also exhibited more frequent ensemble occurrence (p = 0.0268, *t* test; n = 5 fish for each condition: for WT, 3, 1, and 1 fish from 5, 6, and 7 dpf, respectively; for mutants, 2, 2, and 1 fish from 5, 6, and 7 dpf, respectively), and larger ensemble size (p = 0.002, *t* test). Similar increases in frequency of neuronal ensembles were reported in acute hippocampal brain slices from a pilocarpine rodent model of temporal lobe epilepsy (Muldoon et al., 2013) and may be a functional biomarker of a pathological brain state.

Video S3. Optic tectum imaging – WT R representative imaging in optic tectum microcircuits with 20× objective from WT sibling, respectively. Movie played at 40× speed. Related to Figure 7.

Video S4. Optic tectum imaging – mutant Representative imaging in optic tectum microcircuits with 20× objective from *stxbp1b* mutant, respectively. Movie played at 40× speed. Related to Figure 7.

## Discussion

A neural network can exhibit brief periods with elevated spontaneous activity among clusters of interacting neurons. These events have been observed at multiple scales and referred to as neuronal “avalanches” or “cascades.” Here, we analyzed spontaneous brain activity in *stxbp1b* mutant zebrafish larvae using fast confocal imaging techniques in a genetic model of epilepsy and find that cascades are a prominent feature. Interestingly, the size and distribution of cascades observed in *stxbp1b* larval optic tectum were dramatically larger than those observed in controls. These mesoscale events could explain how seizures rapidly propagate in an epileptic brain. Further, as these cascade events were prominent in early neurodevelopment during non-ictal periods, they could represent a functional biomarker of the epileptic brain. Another interesting finding is that cascade neurodevelopmental trajectory was reversed in *stxbp1b* mutants (Figure 5), suggesting cascade activity may be a feature of an epileptogenic process and/or disruption of early development. Taken together, our observations in larval zebrafish are consistent with the description of *STXBP1* as a “neurodevelopmental disorder” (Stamberger et al., 2016).

Previous *in vivo* imaging studies focused on seizure network dynamics were performed using pharmacologically or light-induced activity (Diaz Verdugo et al., 2019; Liao et al., 2019; Liu and Baraban, 2019; Rosch et al., 2018; Turrini et al., 2017; Winter et al., 2017). Whether the latter are true representations of epileptic activity remains to be carefully examined, while the former are best classified as acutely evoked seizure events representing a non-physiological state where inhibitory synapses (by PTZ) or voltage-activated A-type potassium channels (by 4-aminopyridine [4-AP]) are blocked on a global scale. Acute models primarily focus on sporadic ictal events and not the more ubiquitous interictal state experienced by patients with epilepsy. However, this interictal space delineates a potentially more interesting period for developing seizure prediction and/or therapeutic interventions (Gelinas et al., 2016; Huberfeld et al., 2011; Karoly et al., 2016; Tomlinson et al., 2017). In contrast, zebrafish *stxbp1b* mutants recapitulating an epilepsy phenotype seen in *STXBP1* disorder patients exhibit spontaneous whole-brain synchronization (i.e., ictal seizure events confirmed by electrophysiology) at a relatively low frequency. As such, they offer a unique opportunity to study network dynamics during non-ictal periods in a condition free from global pharmacological manipulation. Surprisingly, these interictal periods were marked by abnormally prolonged large cascades in zebrafish *stxbp1b* mutants imaged at a mesoscale level (see Figure 3). LFP recordings from cortical brain slices (Beggs and Plenz, 2003; Shew et al., 2009) describe these cascades as “non-equilibrium states.” A simple interpretation of enhanced cascade activity seen in *stxbp1b* mutants is that they represent a brain state further from equilibrium than normal and thus closer to a disease state defined by “abnormal excessive or synchronous neuronal activity,” (Fisher et al., 2014). Detailed understanding of the crucial cellular elements that drive generation of cascades remains to be determined. That said, a hint provided here and in Ponce-Alvarez (Ponce-Alvarez et al., 2018), using imprecise pharmacological manipulations, is that GJ communication may be critical to cascade generation and/or propagation, which follows an interesting recent observation (Diaz Verdugo et al., 2019), also based on brain-wide imaging in larval zebrafish, that glia-neuron interactions underlie brain state transitions into generalized seizures.

Further evidence of a distinct “epileptic” brain state was seen in microscale analysis of neuronal ensembles (see Figure 7). Here, fast calcium imaging of tectal microcircuits using a transgenic *neuroD1*-promoter line with mosaic single-cell expression provided a high-resolution view of the composition of cascades. Similar to mesoscale analysis, spatially confined neuronal ensembles were noted during interictal periods in epileptic *stxbp1b* mutant zebrafish. Interestingly, similar neuronal ensembles were observed in PTZ-exposed larval zebrafish (Liu and Baraban, 2019), *in vitro* slice preparations from kainic-acid-treated mice (Muldoon et al., 2013), and *in vivo* cortical windows from mice with focally applied 4-AP (Wenzel et al., 2019). These ensembles mimic repetitive, evolving patterns of microdischarges observed in intracranial recordings from patients with epilepsy (Schevon et al., 2008, 2010; Stead et al., 2010). Similar to human microseizures, calcium activity cascades were electrophysiologically distinct from ictal epileptiform events (i.e., spectral hallmark of epilepsy) and localized to isolated microdomains not detected by nearby LFP electrodes. One question raised previously was whether microdischarge events represent abnormal activity related to epilepsy or are simply a feature of the normal brain (Dudek, 2009). Now, incorporating observations made in intact larval zebrafish exposed to chemoconvulsant (Liu and Baraban, 2019) or a genetic form of epilepsy (here), we conclude that while these events are possible under normal wild-type “healthy” conditions, they are distinctly more prominent in the brain of a mutant line confirmed as epileptic. At a network level, one can envision a dynamic range operating at, or near, a balance under normal conditions, oscillating at a functionally disordered state during interictal periods, and then collapsing into to a fully pathological state during an epileptic seizure. Our experimental observations are consistent with the latter explanation.

A more detailed understanding of the critical brain states present in an epileptic brain, both during ictal and interictal periods, underlies our potential ability to design therapeutic interventions. As cell-specific manipulations, optogenetics (Deisseroth, 2011) or designer receptors exclusively activated by designer drugs, DREADDs (Armbruster and Roth, 2005; Armbruster et al., 2007), emerge in parallel to these advanced neuro-imaging technologies, more precisely targeted therapies will become possible. Additionally, as more epileptic zebrafish become available, these concepts can move beyond the single *STXBP1* model observation made here and, perhaps, offer fundamental insights into how the epileptic brain operates.

### Limitations of the study

This study utilized a single zebrafish model representing a genetic form of epilepsy. Analysis was limited to interictal periods where ictal waves were not observed. Whether these observations are generalizable to additional epileptic conditions was not considered. Future studies using multiple zebrafish models could address this limitation. Another interesting possibility to address is whether there is any correlation between activity in the neuronal ensembles and the onset of an ictal-like seizure event. Owing to the low frequency of these events in stxbp1b mutant zebrafish, coupled with relatively brief fast calcium imaging epochs, our studies did not address this question. In addition, the GJ blockers used in these studies are broad and only provide an initial suggestion that electrical communication via GJs play a role in the network activity reported here.

## STAR★methods

### Key resources table

**Table:** 

REAGENT or RESOURCE	SOURCE	IDENTIFIER
**Chemicals, peptides, and recombinant proteins**		

Pentylenetetrazole	Sigma-Aldrich	P6500; CAS: 54-95-5
1-Heptanol	Sigma-Aldrich	820624; CAS: 111-70-6
Propofol	Sigma-Aldrich	Y0000016; CAS: 2078-54-8
Carbenoxolone disodium salt	Sigma-Aldrich	C4790; CAS: 7421-40-1
Mefloquine hydrochloride	Sigma-Aldrich	M2319; CAS: 51773-92-3
Pancuronium bromide	Sigma-Aldrich	P1918; CAS: 15500-66-00

**Critical commercial assays**		

Zebrafish Quick Genotyping DNA Preparation Kit	Bioland Scientific	GT02-01

**Experimental models: organisms/strains**		

Zebrafish: *Tg (neurod1:GCaMP6f)*	C. Wyart. gift	N/A
Zebrafish: *mitfa*^*w2*^	ZIRC	ZL1714
Zebrafish: CRISPR *stxbp1b*^*s3001*^	Grone et al., 2016	N/A

**Software and algorithms**		

MATLAB	MathWorks	https://www.mathworks.com/products/matlab.html

### Resource availability

#### Lead contact

Further information and requests for resources should be directed to and will be fulfilled by the Lead Contact, Scott C. Baraban (scott.baraban@ucsf.edu).

#### Materials availability

This study did not generate new unique reagents.

#### Data and code availability

Raw data were generated in the Baraban laboratory at UCSF. Derived electrophysiology or imaging data supporting the findings of this study are available from the corresponding author upon reasonable request. MATLAB codes used during this study are available upon request.

### Experimental model and subject details

Adult zebrafish were maintained at 28°C on a 14:10 hour light/dark cycle following standard methods. Larvae were raised in embryo media consisting of 0.03% Instant Ocean (Aquarium Systems, Inc.) and 0.0002% methylene blue in reverse osmosis-distilled water. All zebrafish were on a nacre (*mitfa*^*+/-*^) background (White et al., 2008). Heterozygous *stxbp1b* fish were generated by clustered regularly interspaced short palindromic repeats (CRISPR)-mediated knockout, as described (Grone et al., 2016) and in-crossed with transgenic zebrafish expressing neuronal-specific GCaMP6f [*Tg (neurod1:GCaMP6f)* line] (Rupprecht et al., 2016). This unique transgenic *neurod1*:GCaMPf line was developed for fast calcium imaging studies in larval zebrafish (Oldfield et al., 2020). Pigment-free nacre (*mitfa*^*-/-*^) offspring with GCaMP6f expression were sorted on 4 days post fertilization (dpf) and used for calcium imaging experiments on 5-7 dpf. Zebrafish sex cannot be determined until approximately 3 weeks post fertilization (Liew and Orbán, 2014). All procedures followed National Institute of Health and the University of California, San Francisco guidelines and were approved by the Institutional Animal Care and Use Committee (protocol #AN171512-03).

#### Zebrafish genotyping

Animal experiments were performed blind. All larvae were removed from agar at the conclusion of imaging experiments and genotyped for *post hoc* identification of wild-type (WT) and mutant larvae. Briefly, genomic DNA was extracted from whole larvae using the Zebrafish Quick Genotyping DNA Preparation Kit (Bioland Scientific). *stxbp1b* gDNA was amplified using previously described primers (Grone et al., 2016), and then digested with enzyme BsiHKAI at 65°C for 2 hours. Gel electrophoresis (2% agarose) was used to separate digested samples and identify genotype.

### Method details

#### Calcium imaging and LFP recording

All zebrafish larvae were paralyzed in pancuronium (300 μM, Abcam) for 3-5 min, and then restrained in 2% low-melting point agarose in a custom-fabricated recording chamber dorsal side up. The recording chamber was mounted on Zeiss Axiocam upright microscope equipped with Yokogawa Spinning Disk Confocal and a 470 nm laser light source (LaserStack, 3i Intelligent Imaging Innovations). The recording chamber was filled with embryo media containing pancuronium to minimize movement artifact during imaging experiments. After at least 15 min habituation, data acquisition was performed using 5x and 20x objectives. Images were acquired at 20 frames per second (fps) with an EMCCD camera (Photometrics Evolve) at a single plane encompassing telencephalon, optic tectum, cerebellum and hindbrain regions in the field of view. Multiple 5 min recording epochs were acquired for each experiment using SlideBook software (3i Intelligent Imaging Innovations; n = 2∼3 imaging epochs obtained per fish; n = 28 fish recorded). Simultaneous local field potential recordings with an extracellular microelectrode placed in optic tectum or midbrain were obtained, as described previously (Liu and Baraban, 2019). Electrodes were filled with 2 M NaCl, and LFP was recorded using an Axopatch 1D amplifier (Molecular Devices). Signals were lowpass filtered at 1 kHz (–3 dB, 8-pole Bessel), high-pass filtered at 0.1 Hz, digitized at 10 kHz using a Digidata 1520 A/D interface, and stored on a PC computer running Axoclamp software (Molecular Devices). Imaging epochs where an ictal-like seizure event - defined as an electrical event greater than 5x baseline noise, multi-spike and > 500 ms in duration (Griffin et al., 2019) - was detected in the LFP were excluded from further analysis.

#### Pharmacological experiments

Gap junction blockers heptanol (500 μM, Sigma-Aldrich) (Guan et al., 1997; Johnston et al., 1980; Weingart and Bukauskas, 1998) or propofol (10 μM, Sigma-Aldrich) (Mantz et al., 1993; Wentlandt et al., 2006) were added to the bath for at least 30 min to allow adequate diffusion before imaging acquisition. Additional putative gap junction blockers were also tested in preliminary studies but were difficult to dissolve in embryo media (carbenoxolone) or toxic (mefloquine) and not included here. For all drugs, toxicity tests were performed wherein multiple concentrations of each drug were bath applied to 3 agar-embedded larvae for 1.5 hours, then heart rate was monitored to identify maximum non-toxic concentration of each drug to be used. During all imaging studies, heart rate was continuously monitored as a means to confirm vitality, and fish with no or barely observable heart activity after imaging data acquistions were excluded from analysis.

### Data analysis

#### Image processing

Upon sudden exposure to Laser ON light at the outset of imaging data acquisition epochs we noted a brief neuronal GCaMP response and chose to discard the first 5 seconds (100 frames) from all image acquisition data sets. Images were processed for motion correction using the NoRMCorre algorithm (Pnevmatikakis and Giovannucci, 2017) in MATLAB (MathWorks). Regions of interest (ROIs) for optic tectum or single neurons were manually segmented using ROI manager feature in ImageJ. ROI segmentation files were imported to MATLAB for fluorescence signal extraction and analysis. For each pixel (2.67 x 2.67 μm area for whole-brain imaging with 5x objective), the fluorescence changes (ΔF/F) were calculated by subtracting each data point with the mean of lower 50% of values within previous 10 s sliding window and normalized to the mean of the lower 50% of values within previous 10 s sliding window. Here we used the sliding window method to eliminate global fluorescence drifting during the recording period, and set lower 50% values as a baseline for normalization to avoid generation of artifacts. Fluorescence signals for single neurons were obtained by averaging all pixels within the ROI.

#### Cascade detection

Detection of cascades was performed, as described (Ponce-Alvarez et al., 2018; Scott et al., 2014; Tagliazucchi et al., 2012). As neurons were not resolved at brain-wide scale using the 5x objective, cascades were measured by pixels. The fluorescence signal of each pixel within the optic tectum ROI was first binarized by thresholding ΔF/F with a threshold of 5 times signal standard deviation (+5 SD; Figures 2A and 2B). Above threshold the pixel was set to 1 as active, otherwise it was set to 0 as inactive. As illustrated in Figures 2C and 2D, clusters composed of at least 3 connected coactive pixels were identified in each frame. Cascades were defined as spatiotemporally contiguous clusters of active pixels. A new cascade was initiated with activation of a cluster of active pixels that were not active in the preceding frame, and was continued when there was a spatially contiguous cluster detected in the next frame, and ended when this condition no longer held. Cascade size was given by the cumulative number of pixel activations during a cascade.

#### Ensemble detection

Ensemble events were defined as coactivation of a group of neurons in which a statistically significant number of neurons are active compared with surrogate data sets. Automated event detection was performed using a template-matching algorithm (Schultz et al., 2009). A time-varying correlation coefficient between fluorescence trace and calcium transient templates (from the event waveform library) was calculated. Fluorescence transients with amplitude ΔF/F > 0.05 and correlation coefficient > 0.85 were identified as events. Since the algorithm may sometimes give errors, the event train was then manually corrected by manual deletion of falsely detected events and adding events missed by the algorithm. We used a sliding window to generate a time series of coactivation of neurons by counting the number of events within a 0.5 s (10 frames) window. The binary event data were shuffled 2000 times within neurons, and sliding window counting was performed. Frames with an observed number of coactive neurons > 99.9% of all surrogate values (p < 0.001) were identified as highly active frames with ensemble events.

### Quantification and statistical analysis

We used Student’s *t* test for two-variable comparisons, and Kolmogorov-Smirnov (KS) test for cumulative distribution analysis. Detection of neuronal ensembles was performed in MATLAB by comparing with surrogate data sets (see STAR methods). Individual analyses are described in Results.
